# Exercise training to preserve vitality capacity in ageing

**DOI:** 10.1113/EP091731

**Published:** 2024-11-06

**Authors:** Raymond Jones, Taylor L. Taylor, Robert T. Mankowski, Fitzgerald Dodds, Michael Hankes, Joanna Hobson, Yi Lin, Keith Saffold, Silvienne C. Sint Jago, McKenna A. Tharpe, Emily L. Zumbro, Anna Thalacker‐Mercer, Thomas W. Buford

**Affiliations:** ^1^ Department of Medicine University of Alabama at Birmingham Birmingham Alabama USA; ^2^ UAB Center for Exercise Medicine University of Alabama at Birmingham Birmingham Alabama USA; ^3^ Department of Family and Community Medicine University of Alabama at Birmingham Birmingham Alabama USA; ^4^ Department of Physical Therapy University of Alabama at Birmingham Birmingham Alabama USA; ^5^ Department of Cell, Developmental and Integrative Biology University of Alabama at Birmingham Birmingham Alabama USA; ^6^ Geriatric Research Education and Clinical Center Birmingham VA Medical Center Birmingham Alabama USA

**Keywords:** ageing, exercise, physical function

## Abstract

Ageing is an escalating global health issue, with the World Health Organization (WHO) reporting that one in six individuals will be 60 years or older by the year 2030. Therefore, understanding the mechanisms of complex biological ageing processes and associated healthcare challenges has become increasingly important. Intrinsic capacity (IC), defined by WHO as the composite of all physical and mental capacities an individual possesses, can be used as a proxy for defining healthy ageing. IC has five key components: locomotion, cognition, psychological, sensory, and vitality capacity (VC). This review paper specifically focuses on exercise as an effective tool to preserve VC in ageing populations. The physiological domains of VC discussed include energy and metabolism, neuromuscular function, immune and stress response, mitochondrial function, and the methylation clock. Additionally, we highlight potential outcome measures for assessing each of these domains. This review also covers areas of focus for future research and possible interventions. We ultimately conclude that ageing is a complex, multifaceted process resulting in deficits across multiple VC components. However, regular exercise is capable of producing physiological adaptations that may be beneficial in the context of healthy ageing and improving or preserving the status of VC components.

## Abstract (highlights)

- **What is the topic of this review?**
  Recent information on vitality capacity and the importance of using this measure to assess the global effects of exercise training by linking physiological mechanisms induced by exercise training to improvements in vitality capacity.
- **What advances does it highlight?**
  The importance of assessing vitality capacity among older adults to determine the effectiveness of exercise training. Exercise training impacts multiple physiological systems, which contribute to changes in vitality capacity.

## 1 INTRODUCTION

Intrinsic capacity (IC), as defined by the World Health Organization (WHO), is a key component of functional ability, encompassing locomotion (i.e., state of the musculoskeletal system), cognition (i.e., range of mental processes), and psychological (i.e., state and trait emotional factors), sensory (i.e., vision, hearing, pain) and vitality capacity (VC) (Cesari et al., 2018). Ageing is accompanied by a decline in various components of IC leading to increased dependency in later life (Dhakal & Bobrin, 2023; Veronese et al., 2022).

**FIGURE 1:**
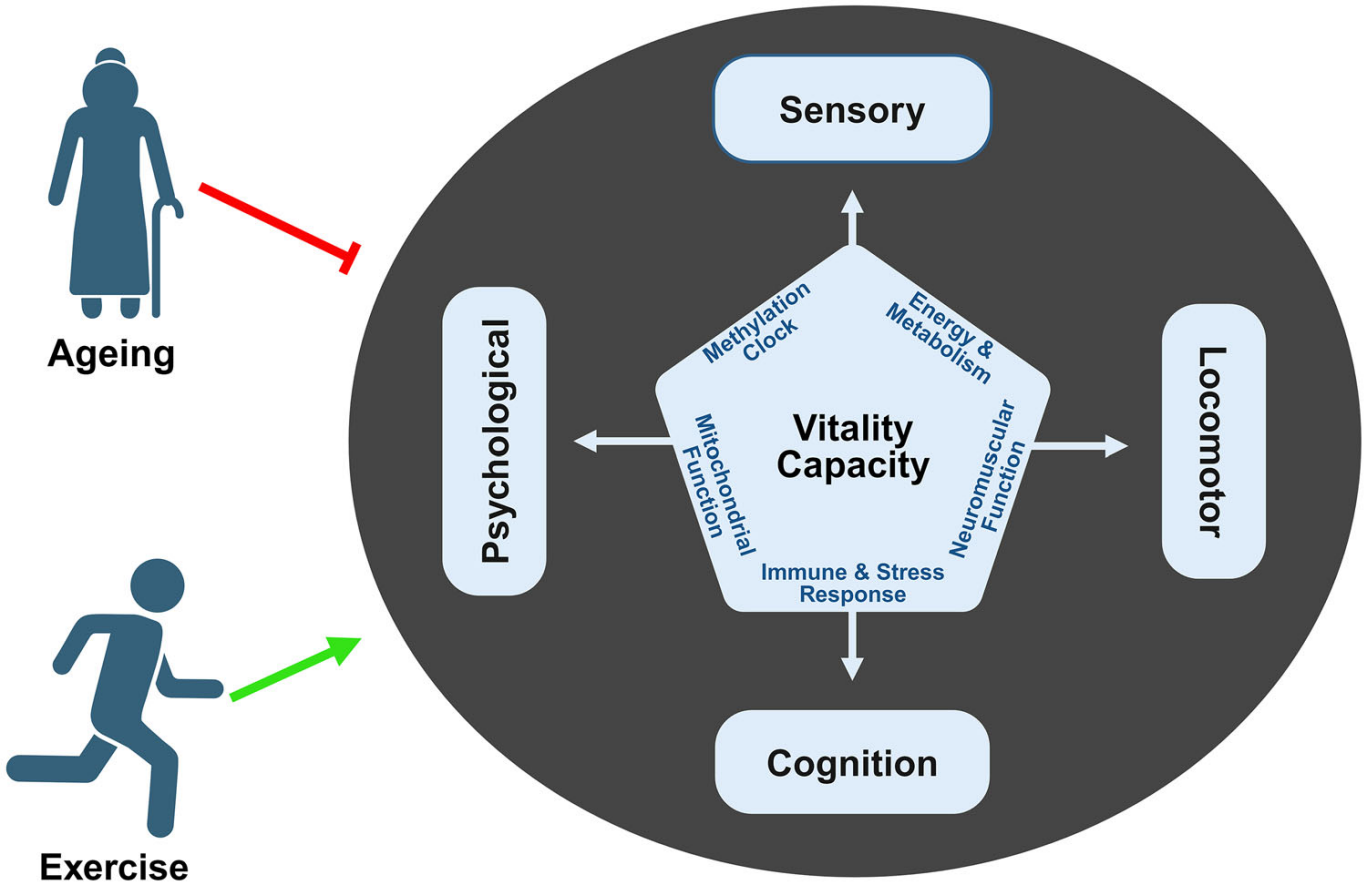
Vitality capacity is the underpinning of other intrinsic capacity components and a modulator of exercise.

VC, distinct from other components of IC, reflects physical and mental vigour and resilience, influenced by changes in energy and metabolism, neuromuscular function, immune and stress response, mitochondrial function, and the methylation clock (Bautmans et al., 2022). While other components of IC focus on performance, VC modulates physical performance, delving into biological systems underlying these capacities (Figure 1) (Beard et al., 2019). In the context of ageing, identifying interventions to preserve and enhance VC is essential for maintaining independence and promoting health in older adults (de Breij et al., 2021).

Exercise training is recognized as an effective intervention for mitigating age‐related declines in physical and mental function, promoting health, and enhancing overall well‐being in older adults (Buford et al., 2013, 2014). Although a few studies have focused on the impact of exercise training or physical activity on IC, the effects on VC are still being investigated (Huang et al., 2021; Sanchez‐Sanchez et al., 2022; Tay et al., 2023). Understanding how different exercise training modalities influence VC is crucial for optimizing exercise training prescription and promoting healthy ageing.

The overall purpose of this review was to consolidate recent information on VC and its relevance in assessing the global effects of exercise training in older adults. We summarize physiological mechanisms linking exercise training to improvements in components of VC. We also review available research and clinical implications of assessing exercise training and VC. By integrating current knowledge in this rapidly evolving field, we hope to provide insights and a conceptual model that can inform the development of tailored exercise training interventions aimed at promoting healthy ageing and preserving VC in older adults. To begin, we review the components of VC while describing the potential role that exercise training has in these components. Finally, we describe the implications for determining the role of exercise training on VC among older adults.

## 2 IMPACT OF EXERCISE TRAINING ON THE PHYSIOLOGICAL SYSTEMS OF VITALITY CAPACITY

VC, a complex physiological state influenced by both normal and accelerated biological ageing, emerges from the interaction of various physiological systems such as energy metabolism, neuromuscular functions, and immune and stress responses (Bautmans et al., 2022). Additionally, measures like grip strength within VC serve as indicators of broader underlying factors, including nutritional, immune and hormonal statuses (Beard et al., 2019; Lu et al., 2023). VC extends beyond mere physical vigour by also encompassing the energy needed for mental and social activities crucial for an individual's overall well‐being. This extension further underlines the critical and complex role of VC in capturing the nuanced aspects of physiological health.

### 2.1 Energy and metabolism

Exercise training‐induced adaptations improve measures associated with the VC systems of energy and metabolism, particularly regarding the perception of fatigue. In the context of mitigating age‐related decrements, exercise training elicits multiple adaptations to attenuate subjective fatigue, particularly through improvements in oxygen uptake and oxygen delivery kinetics through changes in cardiac output, capillary density and mitochondrial biogenesis (Pinckard et al., 2019).

The energy and metabolism systems of VC decline with age, encompassing mitochondrial dysfunction, impaired insulin signalling, increased adiposity and gradual muscle loss (sarcopenia) (Cannataro et al., 2021). These age‐related changes in energy metabolism significantly impact VC, contributing to decreased metabolic flexibility, reduced energy production and impaired substrate utilization. Specifically, impaired insulin signalling results in reduced glucose uptake and utilization by cells, while increased adiposity alters the balance of energy storage and utilization. VC decline contributes to age‐related pathologies such as metabolic disorders, cardiovascular diseases and neurodegenerative conditions. Ageing also reduces physical resilience, leading to increased fatigability and impaired homeostasis under stress. However, exercise elicits pleiotropic adaptations that attenuate these detrimental effects, including enhanced mitochondrial function, improved insulin sensitivity, increased lean body mass, improved metabolic health and reduced adiposity (O'Neill, 2013; Pinckard et al., 2019; Yasuda, 2022; Zurlo et al., 1990). These exercise‐induced adaptations directly address the age‐related declines in energy metabolism. Improved insulin sensitivity enhances glucose uptake and utilization, supporting overall energy metabolism. The increase in lean body mass and reduction in adiposity contribute to a more favourable metabolic profile, improving both resting metabolic rate and metabolic flexibility. Exercise also enhances the activity of key metabolic enzymes, improving the efficiency of energy production and utilization in various tissues (Smith et al., 2018).

The pleiotropic adaptations induced by exercise training attenuate age‐related declines in the VC component, promoting healthy ageing and reducing the risk of various age‐related diseases. By augmenting physical resilience and stress responses, exercise training further contributes to preserving VC. In terms of energy metabolism, this improved resilience manifests as enhanced ability to maintain energy homeostasis under various physiological stressors, improved substrate switching between carbohydrates and fats for energy production, and more efficient energy utilization during physical activities. Exercise also promotes the expression of genes involved in energy metabolism, leading to long‐term improvements in metabolic function (Plaza‐Diaz et al., 2022). The multifaceted impact of exercise training on energy and metabolism highlights its potential as a powerful therapeutic and preventive strategy to maintain and enhance VC across the lifespan. Through these metabolic adaptations, exercise not only mitigates the age‐related decrements in energy systems but also promotes overall metabolic health, contributing significantly to the maintenance of VC with advancing age. This includes improved lipid metabolism, enhanced glycogen storage and utilization, and better regulation of energy balance.

### 2.2 Mitochondrial function

Mitochondrial function impacts overall daily function due to energy availability and is affected by ageing, nutritional intake and physical fitness status. Mitochondrial dysfunction, along with a decline in cardiopulmonary function, contributes to the 4–7 mL kg^−1^ min^−1^ decrease in ${\dot{V}}_{O_{2}\max}$ observed each decade in older adults along with the subsequent reduction in VC to perform daily tasks (Garatachea & Lucia, 2013). Additionally, mitochondrial dysfunction is associated with increased reactive oxygen species (ROS) concentrations, which contribute to DNA damage and increased incidence of apoptosis (i.e., caspase‐dependent and caspase‐independent pathways) and senescence (i.e., p53–p16/p21 pathway) with potential multi‐organ deleterious effects (Droge, 2002; Oka et al., 2008). ROS directly impacts mitochondrial function by targeting mitochondrial DNA (mtDNA) and an associated decrease in activity for mitochondrial complexes I and IV, which are composed of subunits encoded by mtDNA, is observed in older adults, contributing to age‐related changes in metabolism (Peterson et al., 2012). As such, the increased ROS production is associated with low VC (Maynard et al., 2013).

Regular physical activity decreases ROS‐driven damage to DNA and increases mitochondrial turnover and quantity, which directly improves mitochondrial function and effectively reduces the age‐related decline in mitochondrial function and quantity (Barbieri et al., 2015; Hakkinen et al., 2002; Parise et al., 2005; Philp et al., 2021; Romanello & Sandri, 2015; Tarnopolsky, 2009). Importantly, peroxisome proliferator‐activated receptor γ coactivator 1‐α (PGC1α) is upregulated by exercise training and is a strong mitochondrial function modulator. For instance, PGC1α modulates the mitochondrial fusion and fission pathways, stimulates mitophagy, and reduces the activation of proteolytic pathways, reducing the incidence of muscle atrophy (Brault et al., 2010; Jager et al., 2007; Soriano et al., 2006; Vainshtein et al., 2015). However, evidence suggests an impaired upregulation of mitochondrial biogenesis pathways and reduced contractile adaptations following exercise training, in adults >80 years of age, indicating reduced skeletal muscle plasticity compared to younger counterparts (Betik et al., 2009; Hepple, 2014; Kim et al., 2017; Ljubicic & Hood, 2009; Slivka et al., 2008). Therefore, early and continued exercise training throughout the lifespan may be most beneficial to maintain VC via sustained mitochondrial function throughout the lifespan.

### 2.3 Neuromuscular function

Knee‐extensor strength, handgrip strength and respiratory muscle strength have been identified as key measures of neuromuscular function and serve as robust indicators of an individual's neuromuscular health and functional performance (Felicio et al., 2021). Ageing adversely affects these measures through progressive loss of muscle mass and strength, decreased motor unit number and size, and reduced neural drive to muscles. These age‐related changes contribute to declining neuromuscular function and, consequently, diminished VC (Shur et al., 2021). Notably, these markers exhibit increased plasticity in response to exercise training interventions, underscoring the neuromuscular system's adaptability across the lifespan (Huschtscha et al., 2021). The integration of resistance and aerobic exercise training, in a comprehensive programme, is essential for maximizing these neuromuscular adaptations and promoting VC across the lifespan.

These adaptations are underpinned by a complex interplay of processes at various levels. At the molecular level, exercise training activates signalling pathways, such as the phosphatidylinositol‐3‐kinase–protein kinase B–mammalian target of rapamycin axis (Vainshtein & Sandri, 2020), which regulate protein synthesis and muscle hypertrophy, and modulates the expression of myogenic regulatory factors, such as myogenic differentiation factor (MyoD) and myogenin (Drummond et al., 2010), which govern muscle cell differentiation and regeneration. At the cellular level, exercise training stimulates satellite cell activation and proliferation, contributing to muscle fibre growth and repair (Bazgir et al., 2017). The integration of resistance and aerobic exercise training, in a comprehensive programme, is essential for maximizing these neuromuscular adaptations and promoting VC across the lifespan. Further research is warranted to elucidate the precise mechanisms governing exercise‐induced neuromuscular plasticity and to develop targeted interventions for specific populations and clinical conditions.

Exercise training serves as a powerful stimulus for optimizing neuromuscular function and preserving VC throughout the lifespan. Exercise counteracts age‐related neuromuscular declines by maintaining muscle mass, enhancing neural activation, and improving motor unit remodelling, thereby contributing to the preservation of VC. The adaptations induced by exercise training, as reflected by key biomarkers, demonstrate the remarkable plasticity of the neuromuscular system and its potential for adaptation across the lifespan. By understanding the underlying mechanisms of exercise training‐induced neuromuscular adaptations, targeted interventions can be developed to maintain and enhance VC in the face of ageing and disease. Exercise training is a critical component in the preservation of neuromuscular function and, ultimately, the optimization of VC.

### 2.4 Immune and stress response

Stress and immune status are major VC components influencing overall physiological health. For example, chronic stress and inflammation are linked to increased risk of cardiovascular disease with age (Osborne et al., 2020), a consequence of immunosenescence (Pawelec, 2018). Immune function gradually declines with age and is accompanied by systemic chronic low‐grade inflammation termed inflammageing (Lopez‐Otin et al., 2023). Inflammageing is characterized by increased circulating levels of proinflammatory cytokines such as tumour necrosis factor‐α (TNF‐α), interleukins (IL‐1β, IL‐6) and C‐reactive protein (CRP) (Ferrucci & Fabbri, 2018). Furthermore, these adaptations also have important implications for skeletal muscle and bone health in older adults. Specifically, age‐related increases in systemic inflammation have been associated with rapid declines in skeletal muscle mass, muscle strength and bone density, contributing to an increased risk of falls and the development of musculoskeletal disorders (Rodrigues et al., 2022). Age‐associated changes in inflammatory and immunological biomarkers of VC are further exacerbated by a concurrent reduction in physiological stress responses (Dhabhar, 2014; Morey et al., 2015). Extended periods of stress (i.e., chronic stress) promote chronic immunopathological and immunosuppressive responses, increasing the likelihood of systemic inflammation, autoimmune disease, and inefficient combative responses to injury and disease (Dhabhar, 2014). Alternatively, ageing presents a vicious cycle in which chronic stress accelerates immunosenescence and inflammageing and impedes immunoprotective responses to stress (Dhabhar, 2014).

Although results vary by exercise training mode and intensity, structured exercise training interventions have been successful at improving immune function and stress response among older adults (Cornish et al., 2020; Emery et al., 2005; Padilha et al., 2021; Rodrigues et al., 2022; Sellami et al., 2018). For example, acute bouts of high‐intensity aerobic exercise have demonstrated detrimental subsequent immunological responses (e.g., increased secretion of stress hormones like cortisol), while bouts of low‐ or moderate‐intensity exercise seem to promote immune function improvement (e.g., elevation in T‐cell proliferative capacity, increased neutrophil function, and NK cell cytotoxic activity) (Sellami et al., 2018). Alternatively, resistance training also promotes immune cell function and enhances the anti‐inflammatory cytokine profile in musculoskeletal tissues (Cornish et al., 2020). Although, associations between habitual exercise training and improved anti‐inflammatory adaptations of T‐lymphocytes and monocytes are clear (Padilha et al., 2021), careful interpretation of the relevant literature is warranted. Specifically for inflammageing, studies show that aerobic exercise and the combination of aerobic and endurance exercise can improve inflammageing markers such as TNF‐α, IL‐1β, IL‐6 and CRP (Malandish & Gulati, 2023). The current consensus is that participation in a long‐term, moderate‐intensity exercise training programme has beneficial immunological and stress response effects in ageing populations (Padilha et al., 2021; Rodrigues et al., 2022; Sellami et al., 2018). However, more research is needed to differentiate between the positive and negative stress and immunological responses to exercise training and its relation to VC in older adults.

### 2.5 Methylation clock

Epigenetic modification is a modifiable regulation of genetic expression without changing the DNA sequence. DNA methylation is a common type of epigenetic modification characterized by the addition of methyl groups to the genetic code, typically at CpG sites, resulting in the modification of subsequent gene expression or the silencing of the specific gene and reduction in translation. Environmental and biological conditions, such as ageing, influence methylation patterns with maximum lifespan negatively correlated with methylation rate (Crofts et al., 2024). DNA methylation clocks, or age‐associated methylation patterns, have been identified and proposed to be a measure of biological ageing and VC (Horvath & Raj, 2018; Nwanaji‐Enwerem et al., 2018; Wagner, 2017; Zheng et al., 2016) and a predictor of life expectancy (Chen et al., 2016). More precisely, DNA methylation clocks are predictive changes in methylation patterns that occur with age and can slow or accelerate based on disease status and environmental factors. Currently, two predictive DNA methylation clocks exist: the Hannum DNA methylation clock (Hannum et al., 2013), which is only validated for blood, and the Horvath DNA methylation clock, a multi‐tissue biological age predictor (Horvath, 2013). Evidence for accelerated epigenetic age with regard to DNA methylation, in which the biological age may be more advanced compared to the chronological age of the individual, lies in age‐related conditions and diseases including elderly neuropathology (Levine et al., 2015; Marioni et al., 2015), Parkinson's disease (Horvath & Ritz, 2015), and reduced physical and cognitive functions (Breitling et al., 2016; Marioni et al., 2015).

Evidence suggests that regular physical activity has the potential to slow the methylation clock and reverse harmful DNA methylation patterns associated with ageing pathologies. Additionally, physiological measures indicative of VC, such as ${\dot{V}}_{O_{2}\max}$ and grip strength, are negatively correlated with the ageing rate (Jokai et al., 2023). It is estimated that highly fit individuals have approximately a 1.5–2 year lower biological age compared to less fit individuals. However, the effect of childhood physical activity on the DNA methylation clock and biological age in older individuals in addition to the comparison of fit to unfit, sedentary older individuals is unknown. Additionally, lifestyle factors may have a more profound effect on DNA methylation ageing patterns compared to physiological measures and the differential effect on multiple tissues is not fully elucidated (Jokai et al., 2023). Therefore, more research is warranted to study lifelong physical activity effects on DNA methylation patterns in combination with lifestyle factors, such as smoking and sleep status, and the potential to slow or reverse methylation clocks and its relation to VC in multiple tissues.

## 3 IMPLICATIONS (RESEARCH AND CLINICAL) AND FUTURE DIRECTIONS

### 3.1 Using VC to measure the effect of exercise training on successful ageing

A crucial consideration for exercise training is defining meaningful endpoints to evaluate ‘slowed ageing’ and the response of these endpoints to the intervention. Specifically, recognizing the impact of exercise training on factors that support VC as a meaningful endpoint to evaluate the impact of exercise training on ageing is a critical next step. Exercise training, unequivocally, supports healthy ageing and is widely considered one of the primary interventions available for preserving healthspan. Still, despite widespread knowledge of the physiological benefits of exercise training, most studies to date have only evaluated choice outcomes from exercise training interventions. Given the pleiotropic effects of exercise training, such studies may be missing potential benefits of exercise training or conversely overestimating by too narrowly evaluating the outcomes. Therefore, there is need for testing and incorporating global measures such as VC, which would comprehensively assess the effects of exercise training on successful ageing.

### 3.2 Assessing VC in the context of the IC framework and need for VC component score

**FIGURE 2:**
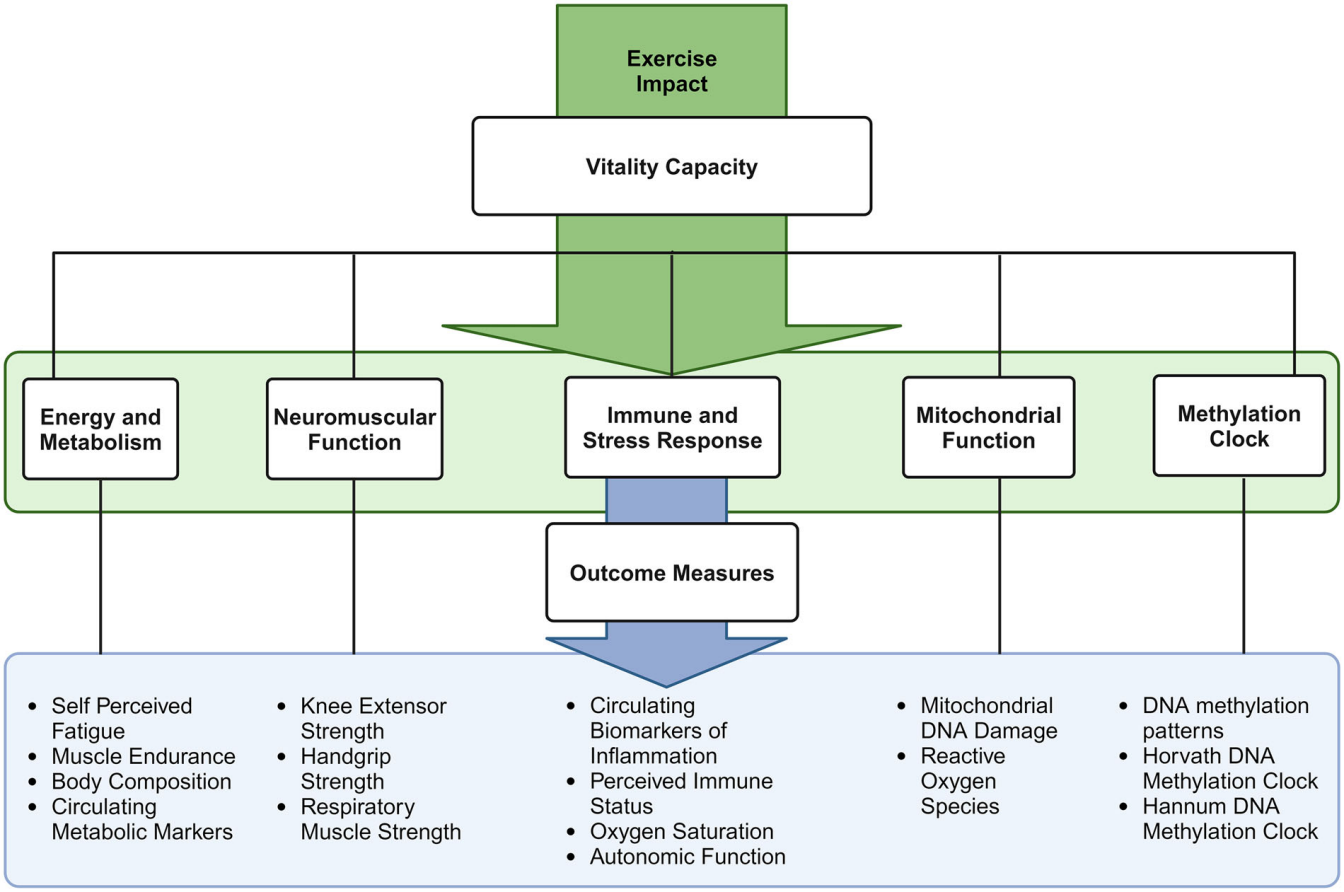
Showcase of the links between exercise measures of vitality capacity to be used as predictors of successful ageing and endpoints for clinical studies.

Because biological ageing is heterogeneous and contributes to a broad understanding of the exercise training response, establishing the potential of a VC test battery can contribute to a more holistic understanding of successful ageing. Within VC, several attributes can be used to provide a score for VC. Previously, researchers used specific measure combinations within the VC component such as blood biomarkers and pulmonary physiology tests (i.e., insulin‐like growth factor 1, dehydroepiandrosterone) and forced expiratory volume). However, given the complex and systemic nature of the ageing process, there is a need for multi‐variable models. Additional measures of VC components such as dietary assessments, handgrip strength, body mass index, body circumference, circulating biomarkers of inflammation and bioelectrical impedance may be used in generating prediction models using cutting‐edge machine learning and artificial intelligence. In the era of bioinformatics, there is a need for a common effort to develop a composite VC score to be used as predictors of successful ageing as well as endpoints for clinical studies (Figure 2).

## 4 CONCLUSIONS

Ageing is a complex process influenced by internal and external factors, interacting with components of VC, which are modifiable via exercise training. We have summarized current evidence supporting the notion that regular exercise training induces multiple physiological adaptations, which contribute to VC modifications. More research is warranted to study the utility of VC, which could be a promising measure of the effects of clinical interventions on successful ageing as a functional model.

## AUTHOR CONTRIBUTIONS

Raymond Jones, Taylor L. Taylor, Robert T. Mankowski, Anna Thalacker‐Mercer, and Thomas W. Buford contributed to the conceptual framework of the review. All authors searched and interpreted the literature. Raymond Jones, Taylor L. Taylor, Silvienne C. Sint Jago, and Emily L. Zumbro were responsible for conceptualizing and designing figures. All authors wrote and revised the manuscript. All authors approved the final version of this manuscript and agree to be accountable for all aspects of the work in ensuring that questions related to the accuracy or integrity of any part of the work are appropriately investigated and resolved. All persons designated as authors qualify for authorship, and all those who qualify for authorship are listed.

## CONFLICT OF INTEREST

None declared.
